# *Zingiber officinale*: A Potential Plant against Rheumatoid Arthritis

**DOI:** 10.1155/2014/159089

**Published:** 2014-05-27

**Authors:** Abdullah Al-Nahain, Rownak Jahan, Mohammed Rahmatullah

**Affiliations:** ^1^Department of Pharmacy, University of Development Alternative, Dhanmondi, Dhaka 1209, Bangladesh; ^2^Department of Biotechnology and Genetic Engineering, University of Development Alternative, Dhaka 1209, Bangladesh

## Abstract

Rheumatoid arthritis (RA) is an autoimmune disease particularly affecting elderly people which leads to massive bone destruction with consequent inflammation, pain, and debility. Allopathic medicine can provide only symptomatic relief. However, *Zingiber officinale* is a plant belonging to the Zingiberaceae family, which has traditionally been used for treatment of RA in alternative medicines of many countries. Many of the phytochemical constituents of the rhizomes of this plant have therapeutic benefits including amelioration of RA. This review attempts to list those phytochemical constituents with their reported mechanisms of action. It is concluded that these phytochemicals can form the basis of discovery of new drugs, which not only can provide symptomatic relief but also may provide total relief from RA by stopping RA-induced bone destruction. As the development of RA is a complex process, further research should be continued towards elucidating the molecular details leading to RA and drugs that can stop or reverse these processes by phytoconstituents of ginger.

## 1. **Introduction**

Rheumatoid arthritis is an autoimmune disease characterized by chronic inflammation due to synovial hyperplasia which further progresses with massive irreversible bone destruction [1–4]. Other symptoms include stiffness and loss of physical movement and systemic features including cardiovascular, pulmonary, physiological, and skeletal disorders [5–7]. Recent epidemiological study shows that about 1% of people all over the world are now affected with rheumatoid arthritis, which exerts significant impact on the quality of life [8, 9]. In all populations, it is more prevalent among women rather than men. Generally, RA is developed (almost in 80% of cases) from the mid of the fourth decade in life to the last of the fifth [10–12]. Medications and lifestyle changes are considered as treatment for RA. Current treatment provides nonsteroidal anti-inflammatory drugs (NSAIDs), for example, salicylic acid and steroids (typically cortisone injection) [13–15]. Though these drugs ease the pain, they are incapable of repairing damaged tissues. Although a broad range of drugs are prescribed for managing the pain and slowing the progression of RA, no drug is known to cure the disease completely [16, 17]. Moreover stomach ulcer is an adverse effect observed in RA patients, regularly taking NSAIDS, and adrenal suppression by steroids [18–20]. These undesirable side effects frequently force the patients to look for complementary and alternative medicine (CAM) [21]. A recent survey indicates that people suffering from chronic pain in RA and those dissatisfied with allopathic treatment are more prone to seek alternative medicine, where 60–90% of arthritic patients use CAM [22, 23]. Therefore, it is highly desirable to find out a potential alternative to eradicate the drawbacks of present allopathic treatment.

Natural products from plants have played a remarkable role to cure and avert different diseases from ancient times [24–26]. A study conducted by World Health Organization (WHO) has reported that about 80% of world's population relies on traditional medicine [27]. In USA, nearly 121 drugs are prescribed today, where 90 of them come from the natural sources particularly from plants in a direct or indirect manner [28].

Herbal remedies can form an alternative source to relieve symptoms in patients having RA as well as to address the drawbacks associated with present treatment methods with allopathic drugs. Among all investigated plants (list of plants is presented in Table 1), it is scientifically palpable that* Zingiber officinale* Roscoe (Zingiberaceae) has a pivotal role to lessen the unbearable pain and inflammation associated with RA [29, 30].

The objective of this present review is to evaluate the therapeutic potential of* Zingiber officinale* in rheumatoid arthritis. We have also aimed to present a summary of mechanism of action of specific phytochemicals of* Zingiber officinale* to reduce the pain claimed by RA-affected patients.

## 2. *Zingiber officinale* in Rheumatoid Arthritis

### 2.1. Pathophysiology of Rheumatoid Arthritis (RA)

**Figure 1:**
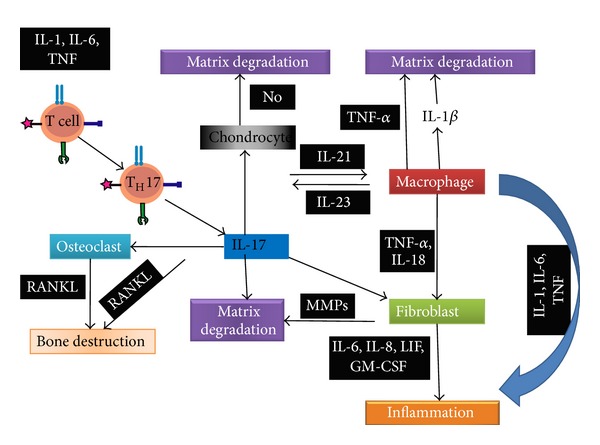
Figure illustrates the pathogenic pathways of rheumatoid arthritis (following [31–38]). As discussed in the text, ginger and its constituents alleviate both bone destruction and inflammation.

Though a great deal of research has been carried out to determine the exact mechanism of inflammation raised in arthritis and consequent tissue destruction, the precise mechanism(s) are yet to be elucidated. On the basis of extensive research on the pathophysiology behind RA, it appears that development of RA follows a series of complex chain of events. Activated T cells express *α*4*β*1-integrin, which binds to vascular cellular adhesion molecule (VCAM) on the surface of venules in inflamed tissues [31]. As a result activated T cells are allowed to penetrate into extracellular fluid by passing through the endothelial wall [31]. Upon encounter with antigen regarding class II major histocompatibility complex (MHC) on an antigen presenting cell, T cells are activated following receiving the second signal via CD28 molecule on the T cell surface. T cells are generated due to influence of cytokines IL-1, IL-6, IL-12, IL-15, IL-23, T cell receptor (TCR), tumor necrosis factor (TNF), and transforming growth factor (TGF) which further differentiate the T cells to T helper 1 (T_H_1) and T helper 17 (T_H_17) [32]. The cytokine IL-17 secreted from the T_H_17 cells then activates macrophage and IL-21, and IL-23 released from macrophage also activates itself. Potency of IL-17 is accelerated via synergy with the effects of tumor necrosis factor [32–34]. In addition, extension of macrophage infiltration into the synovium enhances the severity and progression of RA by secreting IL-1, IL-6, TNF, and matrix metalloproteinase (MMPs) [32]. Recent evidences suggest that the novel cytokines IL-18 and receptor activator of nuclear factor *κ*B (RANKL) are also behind the etiology of the progression of RA [32, 35, 36]. RANKL is a member of TNF superfamily. It is released from activated T cells and highly present in synovial fibroblasts. These cytokines stimulate synovial fibroblasts and chondrocytes in the adjacent articular cartilage to secrete the enzymes. Furthermore, these enzymes degrade peptidoglycan and the cartilage, which subsequently leads to bone destruction [8, 37]. On the other hand after activation by IL-17, osteoclasts induce intrinsic nitric oxide synthase (iNOS) which further participates in bone destruction. Inflammation is developed following activation of fibroblasts. Notably, IL-17 from T_H_17 cell and TNF-*α* and IL-18 secreted from macrophage activate fibroblasts. These fibroblasts then develop inflammation by releasing IL-6, IL-8, LIF, and GM-CSF which produce prostaglandin E2. Besides this, cytokines IL-1, IL-6, and TNF released from macrophage directly can participate in inflammation. The overall mechanisms behind the pathogenic pathways of rheumatoid arthritis (RA) are presented in Figure 1.

Another important mediator to develop inflammation in RA is leukotriene B4 (LTB_4_) having strong attraction to neutrophils [38]. LTB_4_ is derived from arachidonic acid by sequential biochemical reactions of 5-lipoxygenase (5-LO) and LTA_4_ hydrolase. Inhibition of LTB4 receptor activity may be a potential target to protect AR [38].

### 2.2. *Zingiber officinale*

Ginger is obtained from rhizomes of* Zingiber officinale*. The plant belongs to Zingiberaceae family. Since ancient times, it has been widely used as a medicinal herb and spice [39]. Because of containing phytochemical ingredients and as a beneficial therapeutic agent,* Zingiber officinale *has been contributing pivotal roles against a broad range of diseases like asthma, diabetes, stroke, constipation, and others [29, 40]. It is reported that 100,000 tons of gingers are annually produced, and 80% of this is produced in China [41].

### 2.3. Phytochemical Constituents of* Zingiber officinale*

**Figure 2:**
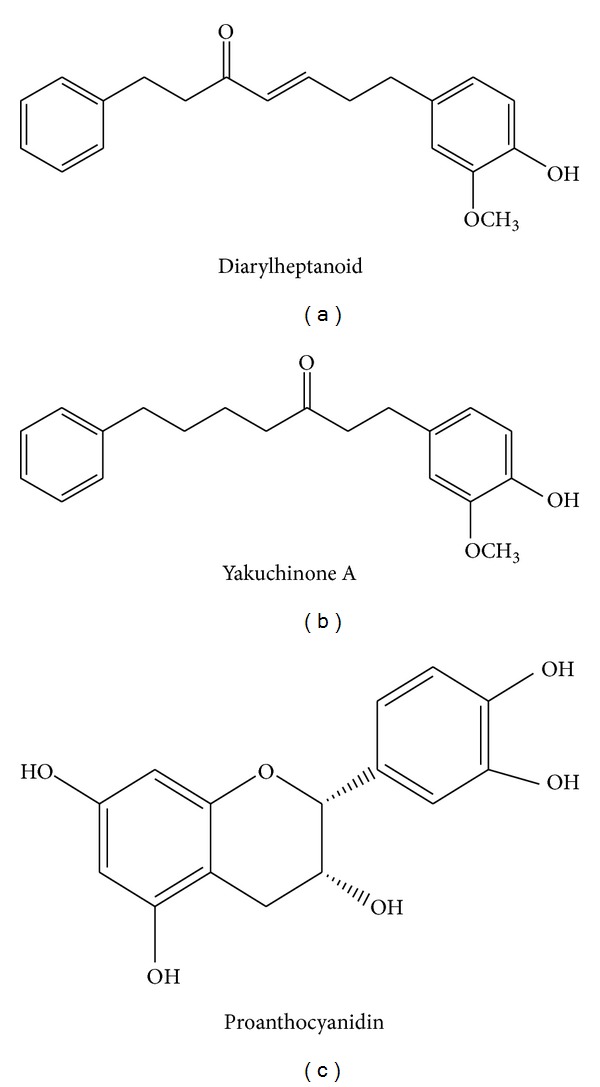
Chemical structures of some phytochemicals of ginger having anti-inflammatory effect.

**Figure 3:**
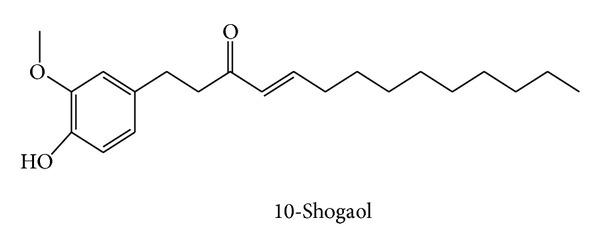
Chemical structure of 10-shogaol.

**Figure 4:**
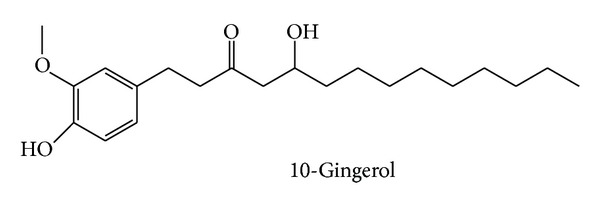
Chemical structure of 10-gingerol.

Ginger contains a large number of phytochemical constituents. The presented table summarizes some major active compounds of ginger. Chemical structures of reported phytochemicals having beneficial effect in RA are presented in Figures 2, 3, and 4.

### 2.4. Beneficial Effects of* Zingiber officinale *on Arthritis Associated Symptoms

Ginger has been cultivated since ancient period as a source of medicinal plant in China as well as other countries all over the world for use as a spice and for therapeutic benefits [42]. Evidences reported that consumption of ginger aids in relieving pain of joints associated with rheumatoid arthritis [43].

Anti-inflammatory effect of ginger was scientifically proved first by Kiuchi et al. in 1982 [44]. They isolated four new different compounds from ginger and all showed potential inhibitory effect to reduce prostaglandin synthesis, which is the key to inflammation. In another study carried out in 1992, they found that ginger showed anti-inflammatory activity by inhibiting not only prostaglandin but also leukotriene biosynthesis [45]. A diarylheptanoid having catechol group showed activity against 5-lipoxygenase which further inhibited leukotriene biosynthesis which can produce an anti-inflammatory effect. Another constituent, namely, yakuchinone A, inhibited prostaglandin production, which can again result in an anti-inflammatory effect.

The activity of* Zingiber officinale* as an anti-inflammatory agent was investigated by Thomson and his group in rats [43]. Experimental rats were treated with aqueous extract of* Zingiber officinale* either orally or intraperitoneally daily for 4 weeks. Though at low dose ginger did not reduce prostaglandin E2 concentrations, at high doses it significantly lowered PGE2 levels. Therefore, ginger could reduce inflammation associated with RA.

Recently,* in vitro* anti-inflammatory effect of ginger was carried out by Ribel-Madsen et al. where they isolated synovial cells from synovial membrane or synovial fluid [46]. Cells were stimulated by TNF-*α*. Ginger treated cells showed similar inhibitory effect to betamethasone by inhibiting production of cytokines IL-1 and IL-6 indicating anti-inflammatory effect.

It is known that 5-lipoxygenase is one of the major key elements of inflammation, and reduction of this factor aids in reducing inflammation. Flynn et al. reported that gingerol and gingerdione significantly showed analgesic and anti-inflammatory activities by inhibiting PGE2 synthesis [47].

Acetic acid induced writhing and foot pad edema tests were evaluated as acute and chronic inflammatory model by Shimoda et al. in experiments with ginger [48]. Considerable suppression in the frequency of writhing and footpad edema primarily indicated anti-inflammatory effect of* Zingiber officinale*. They also determined the effect of prostaglandin and nitric oxide production in mouse leukemic monocytes (RAW 264 cells) stimulated by lipopolysaccharide to find out the mechanism. They demonstrated that both nitric oxide (NO) and prostaglandin production were considerably reduced by the extract of ginger. To find out the responsible active constituents, bioassay guided separation was performed and they concluded that 6-shogaol, gingerdiols, and proanthocyanidins were responsible for these novel effects.

Young et al. observed both analgesic and anti-inflammatory effect of 6-gingerol, one of the major phytochemical constituents of* Zingiber officinale* [49]. Acetic acid writhing and formalin induced licking tests were carried out to evaluate anti-inflammatory effect whereas carrageenan induced paw edema experiment was run to observe analgesic effect in male ICR mice. Both analgesic and anti-inflammatory effects of ethanol extract of dried* Zingiber officinale* were observed by Ojewole [50]. Hot plate and acetic acid tests were carried out in mice to evaluate analgesic effect while egg albumin-induced pedal edema in rat was developed to observe anti-inflammatory effects. Findings from these experimental animal studies indicated potential analgesic and anti-inflammatory activity by ginger which can be applied to reduce pain and inflammation arising from arthritis [50].

Antiarthritic activity of crude extract ginger from* Zingiber officinale* was evaluated in animal model of rheumatoid arthritis and streptococcal cell wall induced arthritis by Funk's group [51]. Therapeutic potency of crude extract was compared with the phytochemical constituent gingerol and its derivatives. They found that the individual phytochemicals showed considerable effect. Interestingly, the crude extract containing essential oils and more polar compounds exhibited better activities by preventing joint inflammation and bone destruction. They concluded that not only gingerol but also non-gingerol compounds of* Zingiber officinale* had considerable antiarthritic activity. Another study by Sharma's group showed both antiarthritic and anti-inflammatory strong effects of ginger oil [52]. In the study, ginger oil was administered orally at a dose of 33 mg/kg for 26 days in arthritic rats. The ginger oil significantly suppressed both paw and joint swelling. Antiarthritic activity by ginger was also observed by Srivastava et al. in patients experiencing rheumatoid arthritis, osteoarthritis, and muscular discomfort independently [53]. More than three-quarters of RA patients of the study experienced relief in pain and swelling remarkably. Surprisingly all the patients of muscular discomfort claimed reduction of pain after receiving ginger powder. This study showed that the beneficial effects of ginger to reduce pain associated with RA were due to inhibition of prostaglandin and leukotriene biosynthesis.

van Breemen et al. reported the mechanism of anti-inflammatory effect of ginger constituents. They found that 10-gingerol, 8-shogaol, and 10-shogaol strongly inhibited COX 2 and thereby significantly reduced inflammation [54]. Though most papers show that ginger exhibits its anti-inflammatory effect by blocking COX-2 enzymes, Grzanna has reported blocking the activities of both COX-1 and COX-2 [55]. Along with these, this study also showed that ginger could suppress leukotriene biosynthesis by inhibiting 5-lipoxygenase [55]. Inhibition of COX-1 activity by ginger was also demonstrated by Nurtjahja-Tjendraputra et al. Their study revealed that 8-paradol of ginger was a potent COX-1 inhibitor [56].

Earlier we mentioned that macrophage plays the pivotal role in the development of arthritis by manufacturing different cytokines and chemokines. If it is possible to inactivate the macrophage, then inflammation can be reduced. Tripathi and her group studied the effectiveness of ginger regarding this condition [57]. Interestingly a positive result was observed where they found that proinflammatory cytokines (IL-12, TNF-*α*, and IL-1*β*) and proinflammatory chemokines release was significantly inhibited in LPS-induced macrophages by the constituents of* Zingiber officinale* [57].

Suppression of inflammation associated with RA by ginger extract was also investigated by Phan et al. in human synoviocytes [58]. The study demonstrated significant inhibition of cytokine expression by* Zingiber officinale*.

Lantz et al. reported that gingerols and shogaols of ginger showed significant anti-inflammatory activity [59]. In their study they found gingerols could inhibit LPS-induced COX-2 expression but shogaol could not do so.

Anti-inflammatory activity of ginger was experimentally compared with ibuprofen (a market available anti-inflammatory drug prescribed for arthritic patient) by Haghighi and his group [60]. Ginger and ibuprofen both showed similar anti-inflammatory activities indicating ginger as a potential anti-inflammatory agent.

Analgesic effect of ginger extract was evaluated in 261 patients affected with osteoarthritis of the knee by Yoshikawa's group [61]. Among all patients, 247 (94.6%) patients claimed to have reduction in pain indicating potential analgesic effect due to administration of extract of* Zingiber officinale*.

Mechanism of inhibition of COX-2 by ginger extract was investigated in-depth by Tjendraputra and coworkers [62]. 8-Paradol and 8-shogaol, the major phytoconstituents of ginger, significantly reduced activities of COX-2 enzyme. The structure activity relationship conducted by them in the same study concluded three possible ways through which 8-paradol and 8-shogaol exhibited their effect to COX-2: (I) lipophilicity of the alkyl side chain, (II) substitution pattern of hydroxy and carbonyl groups on the side chain, and (III) substitution pattern of hydroxy and methoxy groups on the aromatic moiety.

Dual activity of 6-shogaol showed itself by suppressing LPS-induced upexpression of iNOS and COX-2 activities in murine macrophages [63]. 6-Shogaol significantly blocked protein and mRNA expression of iNOS and COX-2 which were determined by Western blotting and reverse transcription-PCR analyses.

Eun et al. (2009) evaluated pharmacological effects of 14 phytochemicals isolated from* Zingiber officinale*, using the RAW 264.7 cell line [64]. They observed that 6-shogaol, 1-dehydro-10-gingerdione, and 10-gingerdione significantly decreased LPS-induced nitric oxide production while the first two remarkably reduced iNOS expression. During development of RA high level of nitric oxide and iNOS play pivotal role in cartilage damage and inflammation. So, these compounds may have beneficial effect to reduce the inflammation in RA.

Both* in vivo* and* in vitro* experiments were conducted to evaluate the effect of 6-gingerol as an inflammatory agent by Pragasam et al. Monosodium urate crystal-induced inflammation was developed in mice as a model of gouty arthritis [65]. They found that 6-gingerol significantly reduced the lysosomal enzymes level as well as inhibiting lactate dehydrogenase and acid phosphate. They concluded that these results were clear indication of anti-inflammatory activity of this ginger phytochemical.

During development of rheumatoid arthritis, toll-like receptors (TLRS) play the vital role in inflammation [66]. Cellular activity of nuclear factor *κβ* (NF-*κβ*) was stimulated by TLR ligands through adaptor molecules, like the myeloid differentiation primary-response gene 88 (MyD88) at an early time point, and the toll/IL-1 receptor domain-containing adaptor-inducing IFN-*β* (TRIF) in delayed kinetics [67]. Under normal conditions, NF-*κ*B was sequestered in the cytoplasm as an inactive complex bound to inhibitory *κβ* (I*κ*B) proteins [68]. Cytoplasmic I*κ*Bs were phosphorylated by TLR2/6, TLR4, or TLR5 agonist which stimulated macrophages or other target cells. This phosphorylation was induced by the catalytic activity of I*κ*B kinase b (IKK*β*) [69]. After degradation of phospho (p)-I*κ*Bs by proteasome, NF-*κβ* was translocated into the nucleus [70]. For transcriptional activation of nuclear NF-*κβ*, the promoter regions of inflammatory genes including inducible iNOS, COX-2, and IL-6 bind to the *κ*B motifs [71]. Therefore, NF-*κβ* is an attractive therapeutic target for inflammatory and autoimmune disorders.* Zingiber officinale* can be a potential one which can inhibit the function of NF-*κβ*. For example, Lee et al. demonstrated that 6-gingerol isolated from* Zingiber officinale* exhibited anti-inflammatory effect by blocking NF-*κβ* and protein kinase C (PKC) signaling pathways [72]. 6-Gingerol significantly suppressed Ik*βα* phosphorylation, NF-*κβ* nuclear activation, and PKC-*α* translocation which in turn inhibited Ca^2+^ mobilization and disrupted mitochondrial membrane potential in LPS-stimulated macrophages. Therefore inducible nitric oxide synthase and TNF-*α* expression were significantly inhibited and reduced inflammation. Recently it has been reported that 1-dehydro-10-gingerdione is one of the significant compounds having anti-inflammatory activity by suppressing the NF-*κ*B regulated expression of inflammatory genes linked to TLR mediated innate immunity [73]. They carried this study in RAW 264.7 macrophages or primary macrophages derived from bone marrows of C57BL/6 or C3H/HeJ mice, which were stimulated with the TLR4 agonist LPS in the presence of 1-dehydro-10-gingerdione. The expression of inflammatory genes was assayed by RT-PCR analysis and a promoter-dependent reporter assay and catalytic activity of inhibitory *κ*B (I*κ*B) kinase b (IKK*β*) was determined through kinase assay and immunoblot analysis. It was observed that cytoplasmic IKK*β*-catalyzed I*κ*B*α* phosphorylation in macrophages activated by TLR agonists or TNF-*α* was irreversibly inhibited by 1-dehydro-10-gingerdione. These effects of 1-dehydro-[10]-gingerdione were abolished by substitution of the Cys179 with Ala in the activation loop of IKK*β*, indicating a direct interacting site of 1-dehydro-10-gingerdione. Conclusively, this entire study demonstrated that NF-*κ*B activation in LPS-stimulated macrophages was disrupted and the suppression of NF-*κ*B-regulated gene expression of inducible NOS, COX-2, and IL-6 was evident by 1-dehydro-10-gingerdione.

## 3. Conclusion

In conclusion, various phytochemical constituents of ginger have potential therapeutic roles in amelioration of RA symptoms and even possibly RA itself. It is expected that further elucidation of the molecular mechanisms behind the action of these phytochemicals not only can lead to discovery of new drugs for symptomatic relief of RA conditions like inflammation and pain, but also may make it possible to stop further progress or even reverse the damage caused by RA.

## Figures and Tables

**Table 1:** Some phytochemical constituents of *Zingiber officinale*.

Groups	Examples	References
Phenolic compounds	Shogaols, paradols, and gingerols	[42, 61, 74, 75]
Sesquiterpenes	Bisapolene, zingiberene, zingiberol, sesquiphellandrene, and curcurmene	[42, 61, 75]
Vitamins	Thiamine, riboflavin, niacin, pyridoxine, vitamin-A, vitamin-C, and vitamin-E	[40, 76]
Others	6-Dehydrogingerdione, galanolactone, gingesulfonic acid, zingerone, geraniol, neral, monoacyldigalactosylglycerols, and gingerglycolipids	[77–79]
